# Development of a Lower Limb Digital Twin Model for Analyzing Femoral Injuries in Elderly CPC Subjects

**DOI:** 10.3390/biomimetics11090674

**Published:** 2026-09-18

**Authors:** Feng Hu, Kun Liu, Sirui Chen, Yumei Yang, Hongxiang Guo

**Affiliations:** College of Intelligent Manufacturing and Energy Engineering, Zhejiang University of Science and Technology, Hangzhou 310023, China

**Keywords:** femoral impact injury, digital twin model of elderly lower limb (DTM-ELL), car to pedestrian collision (CPC), finite element analysis (FEA), biomechanics

## Abstract

In car–pedestrian collisions (CPC), most studies have focused on injury analysis using youth models. However, the mechanical performance of bones and muscles naturally deteriorates with age. Compared with younger people, the elderly are more vulnerable to bone fracture when subjected to the same impact. In this study, a digital twin model of the elderly lower limb (DTM-ELL) and a digital twin model of the youth lower limb (DTM-YLL) for Chinese 50th-percentile males were developed based on CT images, in which 150 material properties were assigned to the bones according to their Hounsfield units. The digital twin model of the lower limb (DTM-LL) was validated through quasi-static three-point bending simulation and dynamic lateral loading simulation of the knee joint. A family-car lateral impact simulation was used to verify the consistency between the developed model and THUMS (Total HUman Model for Safety). Through collision simulations between each of three car models and the DTM-LL, it was found that the maximum average stress in the femur of the DTM-ELL is 62.64% of that of the DTM-YLL under the same CPC conditions. Surrounding soft tissues, acting as effective cushioning during CPC, reduce the maximum femoral stress by 29.56% relative to the model without surrounding soft tissues. Finally, a real CPC accident was reconstructed in finite element software using the DTM-LL developed from the CT images. The reconstruction results showed that the DTM-LL had acceptable biofidelity and can be used to study femoral injuries in elderly pedestrians involved in CPC.

## 1. Introduction

In recent years, with the rapidly increasing number of vehicles, the pedestrian fatality rate caused by traffic accidents has risen year by year [1]. The World Health Organization (WHO) reports that road traffic injuries claim more than 1.2 million lives every year, among which vulnerable road users (pedestrians, cyclists and motorcyclists) account for almost half of these fatalities [2]. In car–pedestrian collisions (CPC), 85% of pedestrians suffer lower limb injuries [3]. According to a survey [4], around eight million hip fractures are expected to occur annually in the coming years. Unfortunately, within six months after a hip fracture, only about one-third of patients recover to their pre-injury functional level, resulting in a significant financial burden on society and families [5]. Compared with younger people, the elderly are more vulnerable to bone fracture when subjected to the same impact because of the reduced mechanical properties of their lower limb bones. For the elderly, age-related deterioration of bone mechanical properties is an important factor affecting the severity of lower limb fractures. The performance of bones and muscles naturally deteriorates with age, for example through bone calcium loss and muscle atrophy [6], which decreases the bones’ tolerance limit and mechanical properties [3]. These changes cause a gradual loss of bone density and a decrease in Young’s modulus, leading to increased brittleness and making the bones more prone to fracture under impact. Therefore, it is of great significance to study impact fractures of the lower limb in the elderly and to analyze the mechanical factors affecting bone fractures, so as to prevent lower limb injuries in this population.

Owing to the difficulty in obtaining Post-Mortem Human Subject (PMHS) experimental samples, researchers have increasingly used finite element analysis (FEA) software to study lower limb bone injuries, owing to its low cost and high simulation accuracy [7]. Moshfeghifar et al. [8] reconstructed 11 healthy subject-specific models, including the sacrum, pelvic bones, proximal femurs, hip joints, sacroiliac joints, and pubic symphysis, providing clinical researchers with free access to verified and reproducible models. In lower limb injury studies, however, Moshfeghifar’s model does not include soft tissues such as skeletal muscle around the bone, which leads to rigid contact between the bone and the impactor. Grindle et al. [9] developed a finite element model including bone, thoracic and abdominal viscera, and passive muscle for the 50th percentile North American male to predict injuries from CPC. Although the model developed by Grindle et al. is a full-body model, it also increases the difficulty of modeling and the computational time of FE simulations when studying lower limb fractures during CPC. Bessho et al. [10] developed femoral finite element models based on axial CT images of three patients with contralateral hip fracture, and predicted the femoral failure location and hip fracture load under two loading conditions: single-limb stance and accidental fall. However, Bessho et al. only predicted fracture locations of the in vitro femur under single-limb stance and accidental fall, without considering the effect of the muscles and other tissues surrounding the femur. In lower limb injury analyses, Haider et al. [11] conducted loading experiments on the femur to study the effect of dynamic loads on the strain field of a single femur under three commonly used boundary conditions, but treating the femur as a separate segment does not reflect its true anatomical state in the human body. Han [12] developed a three-dimensional (3D) finite element model of the human lower limb (HBM-LL) based on human anatomy, which predicts the risk of long bone fractures in the lower limb during traffic accidents with acceptable biofidelity. Mo et al. [13] developed a lower limb–pelvis model with active 3D muscles, and validated the passive and active properties of the muscles against PMHS experiments and volunteers, respectively. Furthermore, finite element analysis has been widely applied in evaluating alternative biomaterials and internal fixation configurations for femoral fractures [14,15]. Mo et al. accounted for the active force contribution of muscles, but during the instant of CPC the muscles play a largely passive role. At present, most lower limb injury studies are based on FEA models of healthy young adults, whereas few researchers have focused on the elderly, who are more severely injured by impacts. There is also a lack of impact injury research based on an elderly digital human body. Moreover, most existing human body finite element models (e.g., the standard THUMS or GHBMC) represent young or middle-aged 50th-percentile males and fail to reflect age-induced biomechanical changes, such as cortical bone thinning, increased porosity, Young’s modulus degradation, and soft tissue atrophy. Consequently, using young models leads to inaccurate injury threshold predictions for elderly pedestrians. Developing elderly-specific lower limb models is therefore critical not only for advancing forensic accident reconstruction, but also for providing biomechanical guidelines for vehicle safety design tailored to aging populations.

In this paper, a more biomimetic digital twin model of the elderly lower limb (DTM-ELL) for estimating femoral impact fracture was developed based on medical imaging, and can be used to study the biomechanical response of the lower limbs of the elderly after impact collisions from an engineering perspective. Based on the principles of multi-body dynamics and lower-limb anatomy, a DTM-ELL with 150 bone material properties was developed according to CT images of the 50th-percentile elderly in China, and its dynamic and static mechanical properties were validated. Furthermore, based on the medical imaging results of a real traffic accident patient with lower-limb femoral injury, the entire CPC process of femoral impact injury was reproduced in the finite element software LS-DYNA (LSTC Corporation, Livermore, CA, USA). Finally, three types of car models (family car, pickup, and sport utility vehicle [SUV]) were used to impact the DTM-ELL laterally at three different speeds of 30, 40, and 50 km/h, respectively, to analyze the kinematics of the femur and its associated impact injury.

## 2. Materials and Methods

### 2.1. Digital Twin Model Development

In this study, the developed lower limb model serves as an offline, subject-specific finite element model (referred to as a computational digital twin in biomechanical modeling) that maps the 3D geometry and bone material distribution of the scanned volunteer without real-time sensor feedback.

This study was performed in line with the principles of the Declaration of Helsinki. Approval was granted by the Medical Ethics Committee of the First Hospital of Jilin University (No. 2023-221). Written and verbal instructions regarding the testing procedures were provided, and informed consent was obtained from the volunteers and the traffic accident victim prior to the experiment.

**Figure 1:**
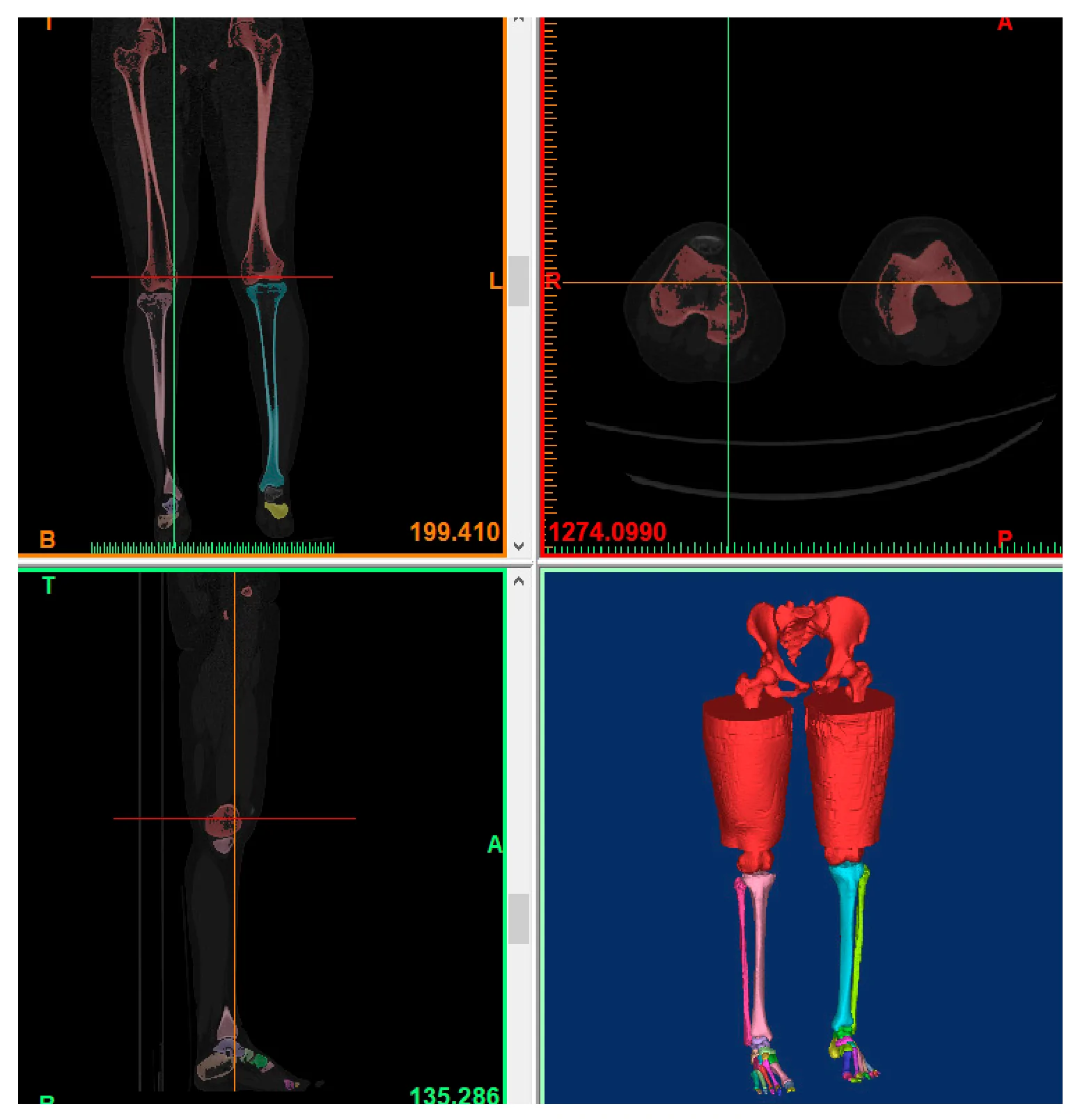
The 3D lower limb model containing bone and soft tissue based on CT images.

**Figure 2:**
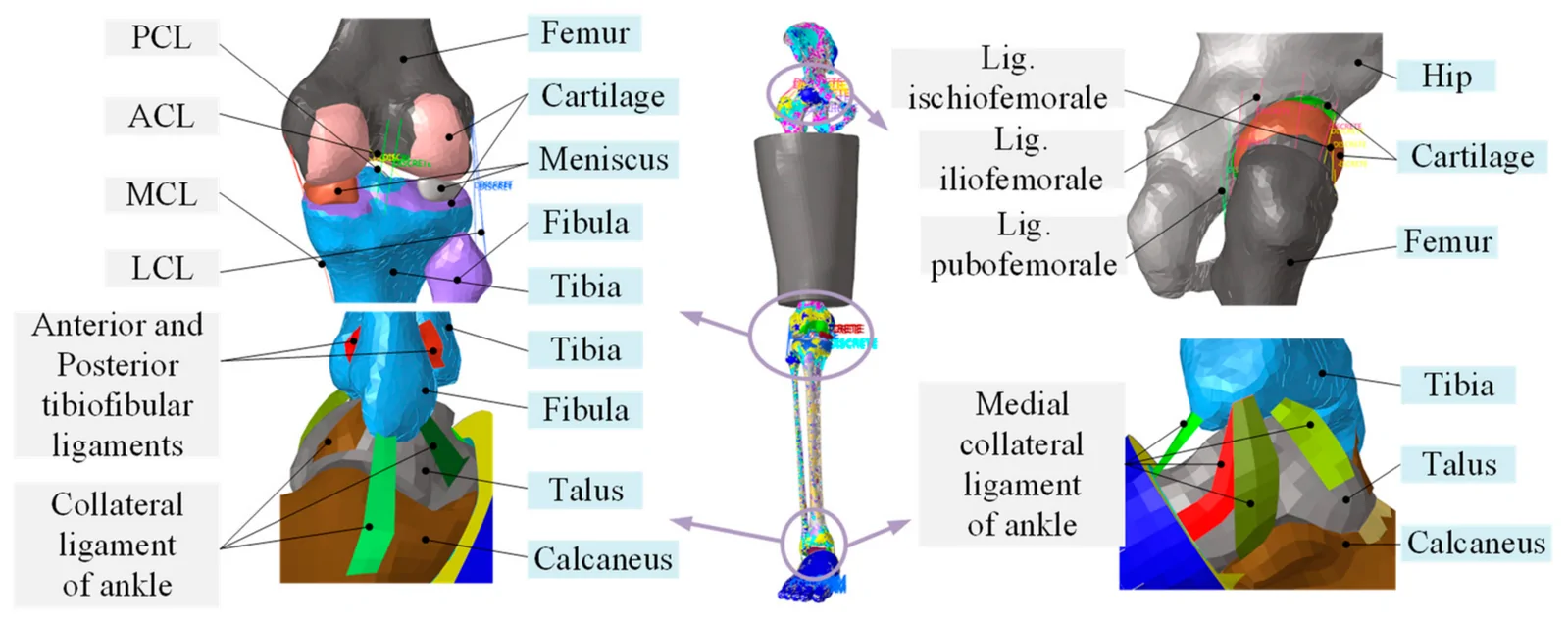
The reconstructed lower limb digital twin finite element model.

According to the human dimensions of Chinese adults GB/T 10000-2023 [16], we identified an elderly male volunteer (height: 165 cm; weight: 65 kg; age: 65 years) whose anthropometry closely matched the 50th percentile of Chinese adult males aged 61–70 years, and a young male volunteer (height: 172 cm; weight: 64 kg; age: 24 years) whose anthropometry closely matched the 50th percentile of Chinese adult males aged 18–25 years. To compare and validate the bone impact fracture performance of the elderly and healthy young volunteers, the lower limbs of both volunteers were scanned using a GE Light Speed QXi scanner (GE Medical Systems, Milwaukee, WI, USA) (120 kV, 250 mA, contiguous 3.0 mm thick slices, 0.313 mm pixels, 512 × 512 matrix, 0.8 s scan pitch), and the corresponding DTM-ELL and DTM-YLL were developed, respectively. Experienced imaging experts evaluated the CT images to exclude the possibility of lower-limb pathological disease in the volunteers. As shown in Figure 1, a 3D lower-limb model containing bone and surrounding soft tissues was developed in Mimics Research 19.0 (Materialise corporation, Leuven, Belgium) based on the volunteers’ CT images, in which the bone was assigned 150 material properties [17] according to the Hounsfield units of the CT images to achieve a more biomimetic bone reconstruction. The lower-limb model was then meshed and the inter-articular constraints were set in HyperMesh 2019 (Altair corporation, Troy, MI, USA) to finally obtain a lower-limb digital twin finite element model, as shown in Figure 2, which contains all the bones of the lower limbs; the hip [18], knee [19], and ankle [20] joints were modeled as bone-to-bone connections through ligaments.

The acetabulum of the hip bone articulates with the femoral head in the hip joint, and the corresponding articular cartilage is contained between them. These bones in the hip joint are connected by hip ligaments, such as the ischiofemoral ligament, pubofemoral ligament, and iliofemoral ligament, which were all modeled as spring units of different bundles with femoral and tibial attachment sites and stiffness values from the cadaver study of Guess et al. [19].

Similar to the hip joint, the distal part of the femur faces the proximal part of the tibia, and the meniscus is modeled on the tibial plateau to form the main structure of the knee joint, which is connected by knee ligaments such as the anterior cruciate ligament (ACL), posterior cruciate ligament (PCL), medial collateral ligament (MCL), and lateral collateral ligament (LCL). For the ankle joint, the main part consists of the distal tibia, distal fibula, and talus. The distal tibia and fibula are connected by the anterior and posterior tibiofibular ligaments, and the distal tibiofibular plateau is connected to the talus by the medial collateral (deltoid) ligament and the lateral collateral ligament of the ankle. To improve the biomimicry of CPC and avoid rigid contact between the femur and the outer surface of the car, the surrounding soft tissues were reconstructed around the femur to simulate the muscles around the femur acting as a cushion during impact. The surrounding soft tissues (muscles and subcutaneous fat) were reconstructed directly from the volunteer’s axial CT contours to preserve realistic anatomical geometry rather than a simplified uniform cylinder. The soft tissues were meshed using 3D solid tetrahedral elements. To simulate the passive cushioning during impact, a simplified foam/soft-tissue material model was assigned with a mass density of ρ = 1.05 g/cm^3^, a bulk modulus K = 1000 MPa, and a passive damping coefficient μ = 0.3, as specified in Table 1. Finally, the lower-limb model was obtained, comprising 144,475 elements and 44,481 nodes. The reliability of the FEA model was ensured by controlling element parameters such as the aspect ratio and element quality. To model the dynamic response of the femur, the plastic–kinematic material model was assigned to the cortical and trabecular bone. Although bone is fundamentally a quasi-brittle material with negligible post-yield strain hardening, this material model, combined with progressive element deletion, effectively captures the elastic response, initial yield, and immediate dynamic fracture under high-rate lateral impact. Owing to this quasi-brittle characteristic, an idealized elastic–perfectly plastic representation was adopted, in which a single effective stress threshold (σy = σu = 116 MPa), adopted from the validated literature [21,22], was used to represent both the baseline yield stress and the nominal ultimate stress threshold across the 150 mapped material bins. Because of the short impact duration (20 ms) in pedestrian collisions, strain-rate dependence was not explicitly modeled in this offline formulation, which is recognized as a methodological limitation and discussed in Section 4.4.

In order to more accurately study the fracture mechanism of the femur, we used a combined criterion based on failure strain and ultimate stress to delete elements and simulate bone fracture. The cortical and trabecular bone were distinguished according to their Young’s modulus, and their failure strains were set accordingly. The material parameters of each part of the lower limb are shown in Table 1 [23,24].

The progressive bone fracture and element elimination were numerically implemented using the *MAT_ADD_EROSION keyword in LS-DYNA. Element deletion was governed by a combined failure criterion: an effective ultimate von Mises stress threshold (σu = 116 MPa) and maximum principal failure strain (εmax = 0.015 for cortical bone; 0.01 for trabecular bone). Under lateral impact bending, element deletion is predominantly initiated on the tensile side of the femoral shaft where the maximum principal tensile strain exceeds εmax, correctly reflecting the quasi-brittle tensile failure characteristics and lower tensile tolerance of cortical bone compared to its compressive strength.

**Table 1:** Definition of material properties in model components.

Part	Material Type	Material Properties
Femoral cortical bone (Youth) [21]	Plastic–kinematic	ρ ϵ (0.8, 2.0) g/cm^3^, E ϵ (4028, 17,815), μ = 0.3, σy = 116 MPa, εmax = 0.015.
Femoral trabecular bone (Youth) [21]	Plastic–kinematic	ρ ϵ (0, 0.8), E ϵ (0, 4028), μ = 0.3, σy = 116 MPa, εmax = 0.01.
Hip, Tibia, Fibula and Foot bones (Youth)	Elastic	ρ ϵ (0, 2.0) g/cm^3^, E ϵ (0, 18,126), μ = 0.3.
Femoral cortical bone (Elder) [22]	Plastic–kinematic	ρ ϵ (0.63, 1.72) g/cm^3^, E ϵ (3439, 15,224), μ = 0.3, σy = 116 MPa, εmax = 0.015.
Femoral trabecular bone (Elder) [22]	Plastic–kinematic	ρ ϵ (0, 0.63) g/cm^3^, E ϵ (0, 3439), μ = 0.3, σy = 116 MPa, εmax = 0.01.
Hip, Tibia, Fibula and Foot bones (Elder)	Elastic	ρ ϵ (0, 1.72) g/cm^3^, E ϵ (0, 15,224), μ = 0.3.
Cartilage [25]	Elastic	ρ = 1 g/cm^3^, μ = 0.46, K = 5 MPa.
Meniscus [26]	Elastic	ρ = 1 g/cm^3^, μ = 0.49, K = 59 MPa.
Hip and Knee ligament [19]	Spring	Ischiofemoral: 3 × K = 13 N/mm; Pubofemoral: 3 × K = 13 N/mm; Iliofemoral: 4 × K = 50 N/mm; PCL, ACL: 2 × K = 20 N/mm; MCL, LCL: 3 × K = 30 N/mm.
Foot ligament	Fabric	Medial collateral ligament: ρ = 1.1 g/cm^3^, E = 435 MPa, μ = 0.22. Collateral ligament, Anterior and Posterior tibiofibular ligaments: ρ = 1.1 g/cm^3^, E = 75.4 MPa, μ = 0.22.
Surrounding soft tissues	Simplified-foam	ρ = 1.05 g/cm^3^, K = 1000 MPa, ς = 0.1.

The 150 bone material properties were assigned based on CT Hounsfield Units (HU) using a standard two-step empirical mapping algorithm [23,24]. Specifically, the HU values of individual CT voxels were first converted into bone apparent density, which was subsequently mapped to Young’s modulus using power-law relationships established for human long bones [23,24]. The material binning was discretized into 150 property levels. Bone fracture was simulated using a combined failure criterion of ultimate stress (σu = 116 MPa) and maximum principal failure strain (εmax = 0.015 for cortical bone; 0.01 for trabecular bone), accompanied by progressive element deletion once the failure thresholds were reached. To ensure numerical stability and mesh independence without adding unnecessary complexity, a mesh convergence check was performed evaluating mean element sizes of 2, 3, and 5 mm. The peak von Mises stress in the femoral shaft varied by less than 2.8% between the 2 mm and 3 mm meshes, confirming that the 3 mm average mesh size used in this study provides an optimal balance between accuracy and computational efficiency. Furthermore, a sensitivity analysis showed that a 10% variation in bone elastic modulus altered the peak impact force by less than 4.1%, demonstrating model robustness. Numerical stability was rigorously maintained throughout all simulations, with hourglass energy strictly controlled below 5% of total internal energy, negligible mass scaling (<1.0%), and a stable global energy balance.

### 2.2. Methodology of Model Validation with PMHS Experimental Data

#### 2.2.1. Femur Validation Model Against Quasi-Static 3-Point Bending

**Figure 3:**
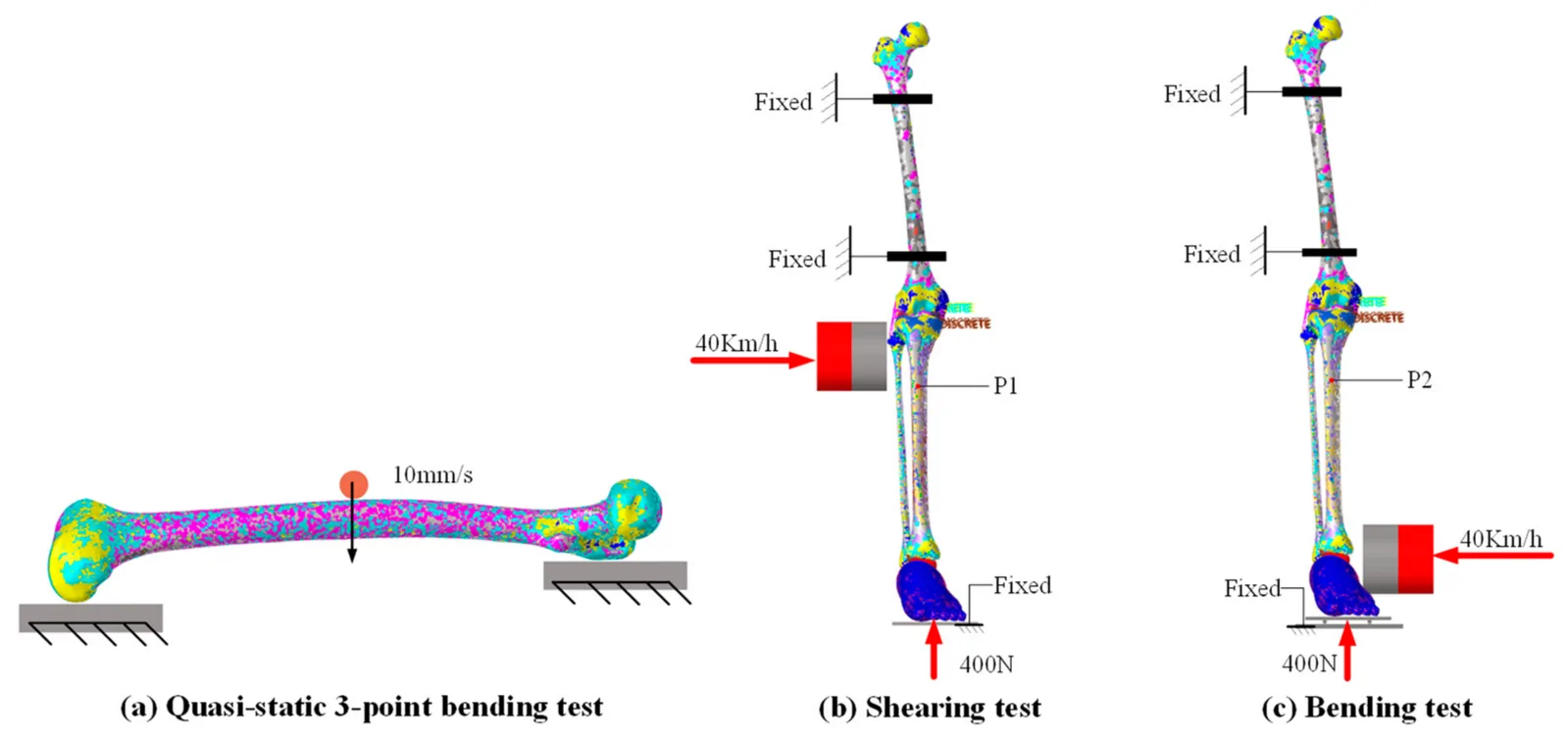
Validation of the DTM-LL.

The PMHS experimental data reported by Yamada [27] were used as the control data to validate the developed digital twin femur model of the young lower limb. As shown in Figure 3a, the distal and proximal ends of the femur were horizontally placed on a support platform, and the femur was then loaded by a bar-shaped indenter with a diameter of 20 mm at a speed of 10 mm/s in a three-point bending test. The changes in the force on the femur with increasing loading displacement were compared with those reported by Yamada [27].

#### 2.2.2. Knee Validation Model Against Dynamic Lateral Loading

In vitro PMHS experiments have shown that both shear deformation and bending deformation of the knee can have important effects on the lower-limb joints. Therefore, corresponding FE validation experiments were designed based on the in vitro experiments of Kajzer [28,29], as shown in Figure 3b,c.

As shown in Figure 3b, referring to the PMHS test conducted by Kajzer [28], a shear test of the DTM-ELL was performed to verify the injury mechanism and tolerance of the knee joint under high-speed lateral impact. The distal and proximal ends of the femur were fixed, and a plantar reaction force of 400 N was applied vertically upward to the sole of the foot. A cylindrical impactor weighing 6.25 kg (r = 50 mm, h = 50 mm) struck the position below the knee joint at a speed of 40 km/h. Styrodur (type: 3035 S, r = 50 mm, h = 50 mm) was attached to the front of the impactor to avoid direct contact between the impactor and the lower-limb model. The ability of the knee joint to withstand shear loads, compared between the DTM-ELL and PMHS, was evaluated by the displacement of the tibial marker point P1 in the direction of impact. Similarly, as shown in Figure 3c, referring to the PMHS test conducted by Kajzer [28], a bending test of the DTM-ELL was performed to verify the injury mechanism and tolerance of the knee joint under high-speed lateral impact. To reduce the effects of friction, the foot was placed on a mobile plate representing the ground. Under the same static operating conditions as the shear test, the same cylindrical impactor struck the ankle region at a speed of 40 km/h. The ability of the knee joint to withstand bending loads, compared between the DTM-ELL and PMHS, was evaluated by the displacement of the tibial marker point P2 in the direction of impact.

### 2.3. Comparison of Biomechanical Responses of the DTM-ELL

To study the biomechanical responses of the DTM-ELL under dynamic impact in CPC, three car models (family car: 1.097 t, pickup: 2.711 t, SUV: 1.51 t) were used as impactors. After a statistical analysis of hospitalized urban road traffic accident patients treated at the First Hospital of Jilin University over the last three months, it was found that the collision speeds of cars in urban road traffic accidents were generally concentrated within 30–50 km/h. Therefore, in the FE simulation analysis, the CPC speed of the car was set to 30, 40, or 50 km/h. According to Yang [30], when a car strikes the lower limb from the lateral side, bending is one of the most important factors leading to long-bone fractures. Therefore, lateral impact was selected for the three cars to observe femoral fracture.

**Figure 4:**
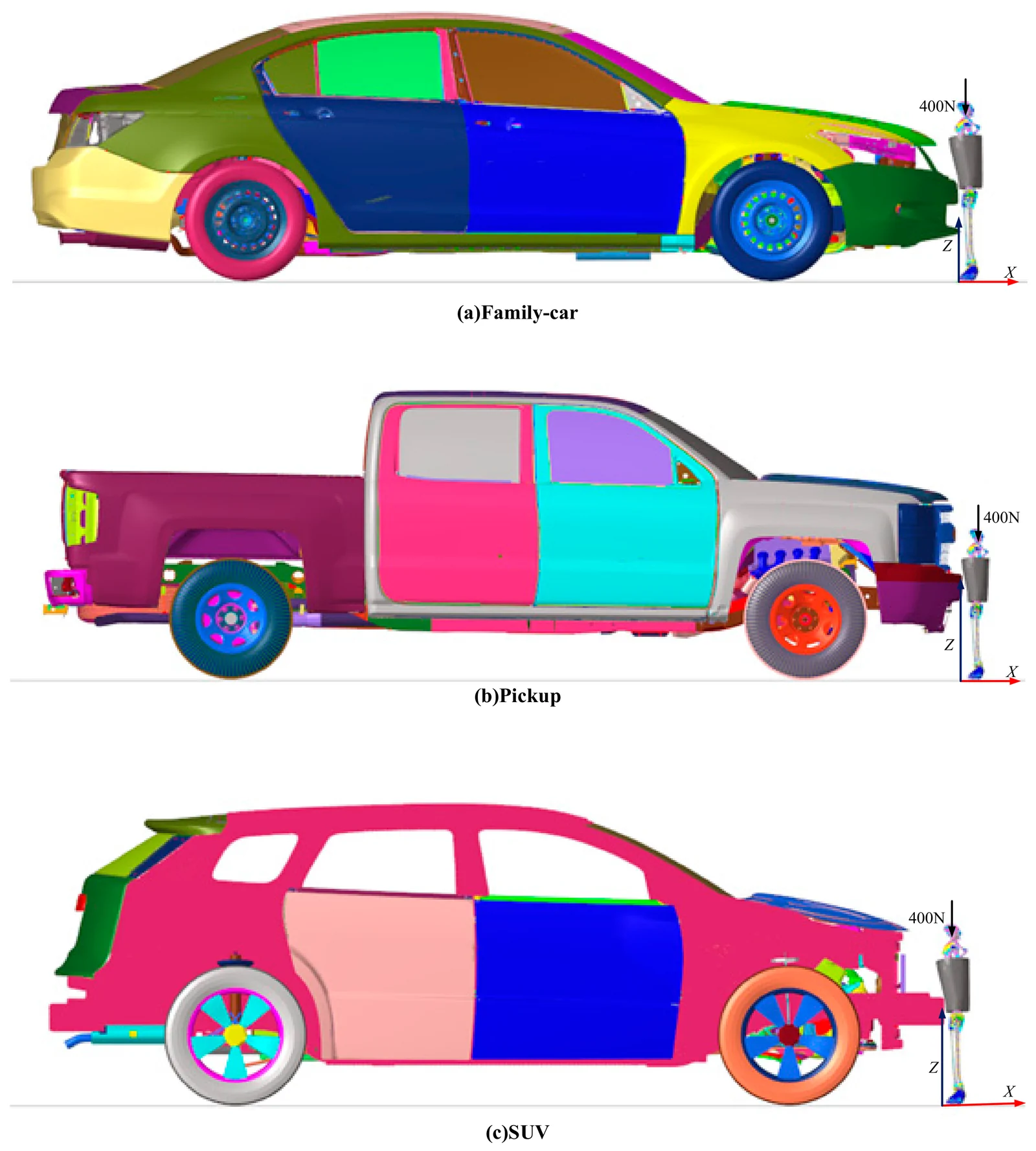
FE simulation of CPC with three types of cars respectively.

As shown in Figure 4, a global coordinate system was established, in which the projection of the front end of the car onto the ground was defined as the origin, the direction of the car’s travel was defined as the positive X-axis, the left-to-right centerline of the car defined the Y-axis, and the vertical direction pointed upward. Each car model impacted the outer side of the pedestrian’s right lower limb at speeds of 30, 40, and 50 km/h, respectively.

The load on the hip bones of the pedestrian from the trunk was set to 400 N, the friction coefficient between the foot and the ground was set to 0.5, and the friction coefficient between the pedestrian and the car was set to 0.3. The simulation duration of 20 ms was chosen based on the physical duration of the primary impact phase during pedestrian lateral collisions, which fully encompasses the dynamic loading and fracture-initiation process. The contact force between the car and the surrounding soft tissues around the femur was defined as the impact force, and the relative displacement of the femur and tibia was defined as the knee shear displacement. The biomechanical responses of the lower limb during CPC impact can thus be observed through the simulation. The effects on lower limb injury of different cars at different velocities were analyzed based on the impact force–time curve and the knee shear displacement–time curve during CPC.

### 2.4. CPC Accident Reconstruction

A case of lower-limb fracture with clear accident information was selected from the traffic accident patients treated at the First Hospital of Jilin University, and the DTM-LL was developed from his CT images for CPC FE simulation analysis using the aforementioned method.

Case description: A 62-year-old man, 165 cm tall and weighing 80 kg, crossed the road at a self-reported fast pace from west to east, crossing in front of a bus. Meanwhile, a car weighing 1.5 t traveling at velocity v was moving beside the bus from south to north in the same direction. The driver braked after seeing the pedestrian and made an emergency right turn to avoid striking him. Nevertheless, the pedestrian was still hit and thrown by the impact, and then stopped in front of the car. In this accident, both the pedestrian and the car were in motion. Therefore, at the moment of impact, the velocity of the car relative to the pedestrian was set to v, and the angle between the velocity direction and the sagittal plane of the lower limbs was θ. After the accident, an X-ray examination showed that the elderly man had a wedge-shaped fracture in the upper one-third of the femur. According to the driver’s description, the traveling speed of the car was approximately 30–40 km/h. The relative vehicle impact velocity was set to 35 km/h at an impact angle of 90° relative to the sagittal plane of the lower limb. The reconstruction accurately matched the patient’s clinical X-ray fracture morphology, with failure determined by local strain concentration and crack initiation at 3.1 ms in the proximal one-third of the femur.

## 3. Results

### 3.1. Model Validation Compared with PMHS Experimental Data

**Figure 5:**
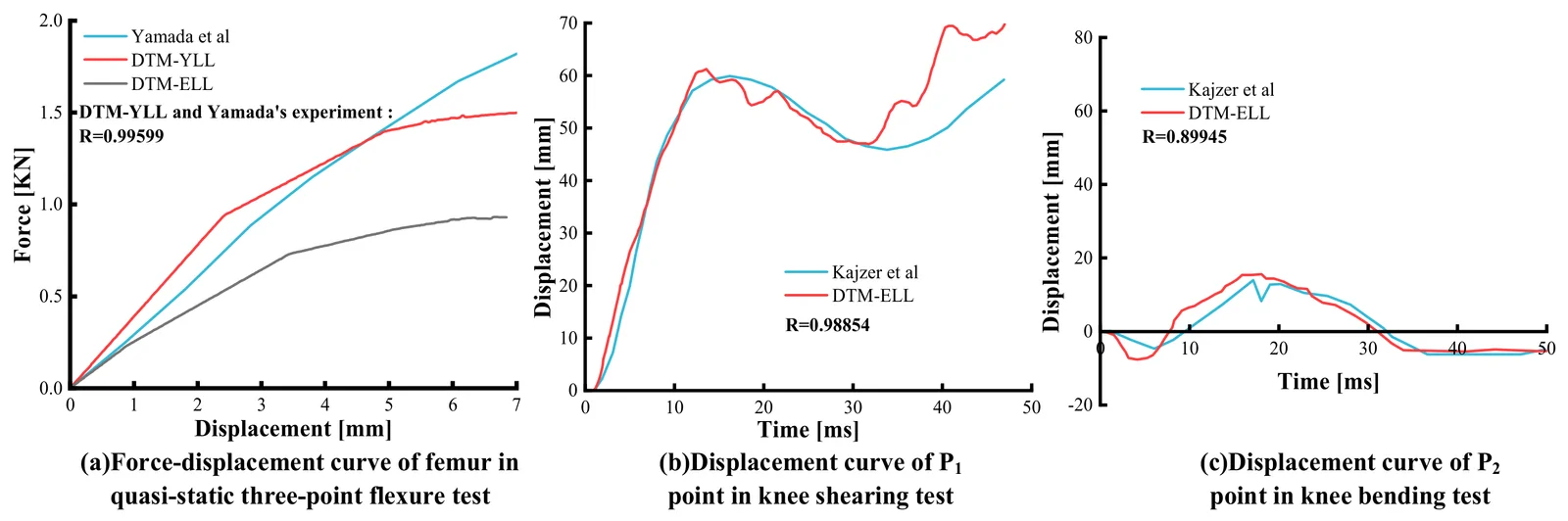
Comparison between the results of FE simulation and control group [27,28].

As shown in Figure 5, the force–displacement curve from the three-point bending experiment of the femur in the DTM-LL shows a strong correlation (R = 0.996) with that of the PMHS data reported by Yamada [27]. Compared with the DTM-YLL, the femoral displacement of the DTM-ELL is greater under the same loads, because the decrease in Young’s modulus causes the bones of the elderly to deform more under the same load.

As shown in Figure 5b,c, the displacement curves of points P1 and P2 in the shear and bending tests of the knee joint both show strong correlations (R = 0.98854 and R = 0.89945, respectively) with those of the PMHS data reported by Kajzer et al. [28]. This indicates that, within the allowable error range, the DTM-LL developed in this study is accurate and demonstrates good biomechanical fidelity. Nevertheless, some error remains between our simulation results and the control group, which may be due to the influence of the soft tissues around the femur in the PMHS experiments [28], which prevents the ideal fully constrained fixation of the femur used in the FE simulation.

The force–displacement and kinematic responses exhibited high correlation with the published PMHS literature (Yamada [27], Kajzer [28]), with Root Mean Square Errors (RMSE) below 8.5% for all loading conditions. In addition, the cross-model comparison with THUMS demonstrated consistent biomechanical trends. However, it should be noted that direct experimental validation using new cadaveric specimens of the specific subjects was not performed in this study due to ethical constraints, representing a partial validation under published boundary conditions. Furthermore, THUMS serves as a computational benchmark rather than experimental ground truth; because the THUMS version represents a standard adult occupant, lower agreement between the elderly model and THUMS is expected and does not invalidate the elderly model.

### 3.2. Comparison of Biomechanical Responses

**Figure 6:**
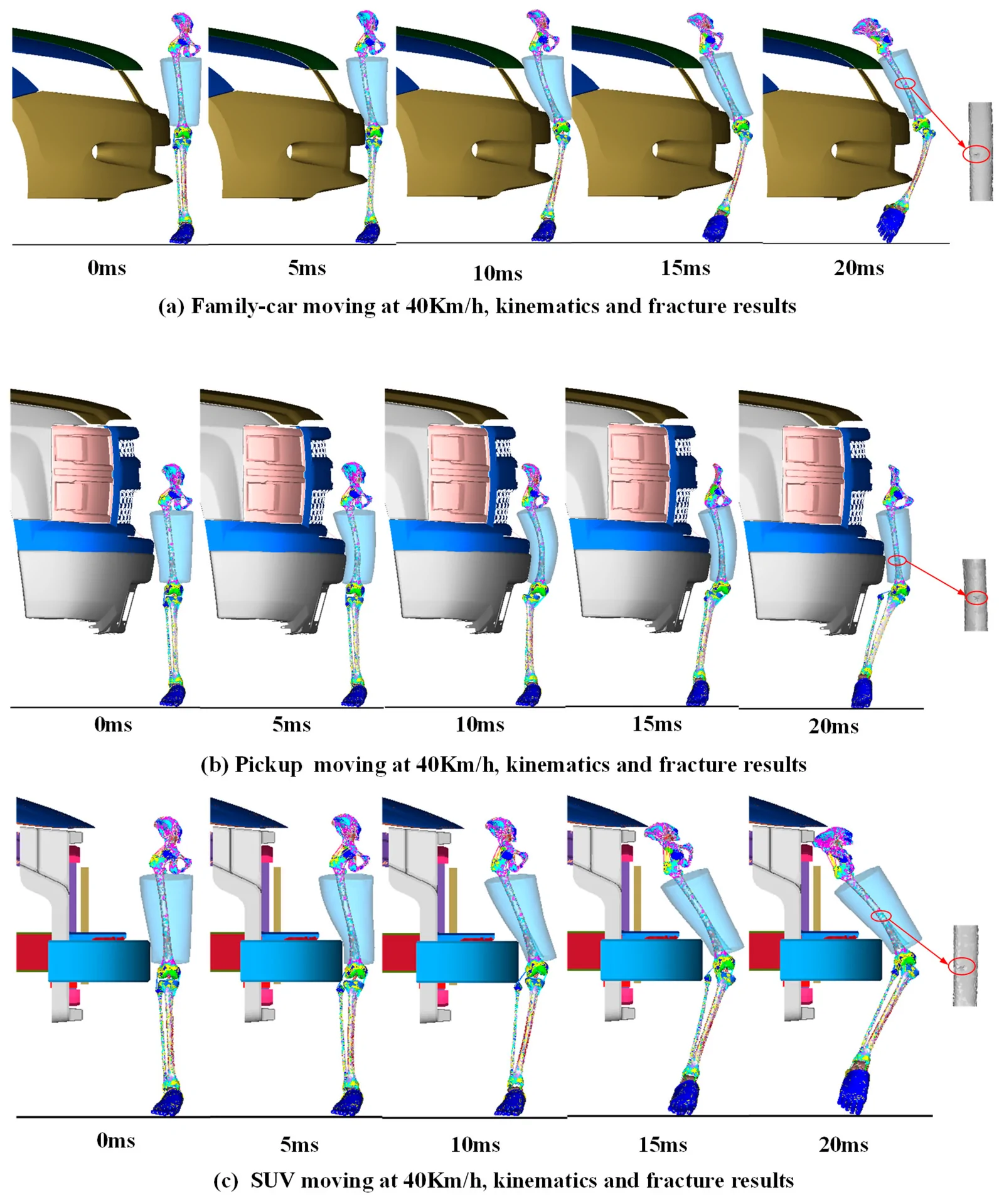
The kinematic response and final fracture location of DTM-ELL impacted by three types of cars at the velocity of 40 km/h.

**Figure 7:**
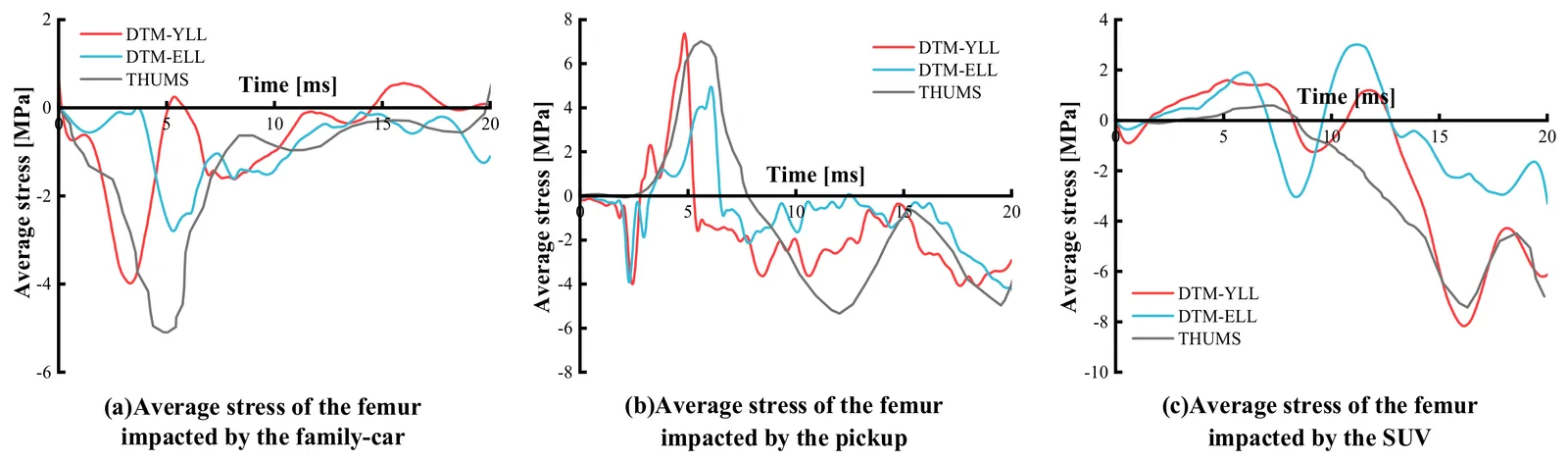
Real-time average stress of the femur in the DTM-ELL, DTM-YLL, and THUMS under lateral impact by three different car types at a speed of 40 km/h.

**Figure 8:**
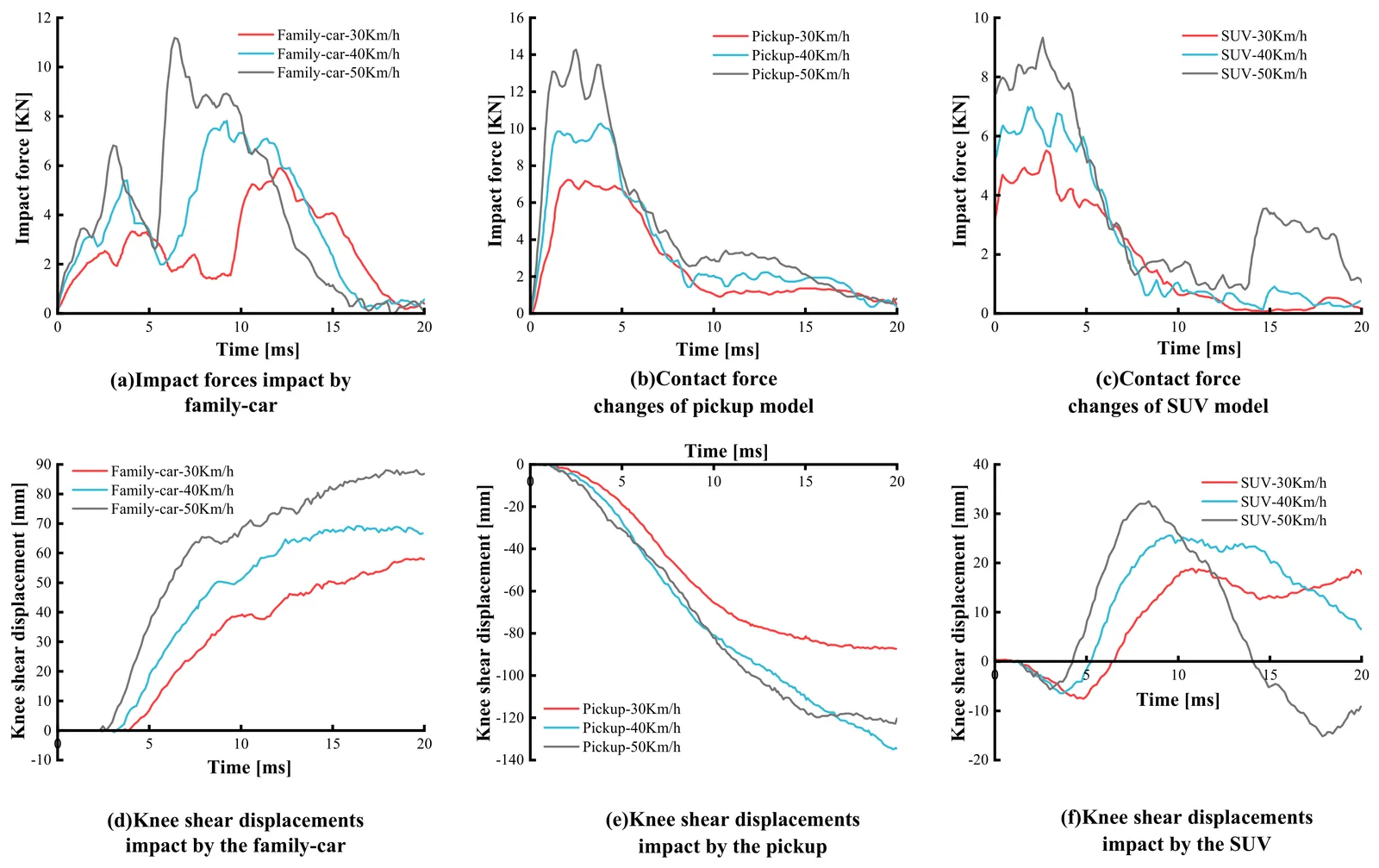
Impact forces and knee shear displacements of the DTM-ELL under lateral impact by three different car types at speeds of 30, 40, and 50 km/h, respectively.

The biomechanical responses, including the kinematic responses and the final fracture location of the DTM-ELL impacted by the three car types at a speed of 40 km/h, are shown in Figure 6a–c. The front bumper of the car contacts the lower limbs at the moment of CPC, and the bones around the impact point move in the opposite direction of the car’s travel relative to the front end of the car because of inertia. The bones above and below the impact point are then struck by the hood and front bumper, respectively, causing the lower limb to bend and deform along the front contour of the car, and resulting in damage to the bones and joint ligaments. The kinematic response of the developed DTM-ELL throughout the entire collision process is essentially consistent with the data published by Han [12], which verifies the correctness of the FE simulation. Furthermore, to verify the correlation between the biomechanical responses of the DTM-ELL and DTM-YLL constructed in this study and those of the control model THUMS (Total HUman Model for Safety, version 4.02), FE simulations of the three models impacted by the three car types at a speed of 40 km/h were performed. In the impacted plane of the femurs of the three models, the stresses of five reference elements along the midpoint of the femoral axis toward the proximal and distal ends were taken every 7 cm, and the average stress of the five reference elements was calculated as the real-time average stress of the femur in each model. The reported real-time average stress and maximum stress refer specifically to the von Mises equivalent stress averaged across five reference solid elements along the femoral shaft axis. The von Mises equivalent stress was selected to evaluate the overall multiaxial stress state under combined bending and shear during impact. In Figure 7, the real-time average stress of the femur in the DTM-ELL, DTM-YLL, and THUMS under lateral impact by the three different car types at a speed of 40 km/h is shown. It can be seen that the real-time average stress of the femur in the DTM-YLL is strongly correlated with that in THUMS under impact by the family car (Rfamily-car = 0.82), the pickup (Rpickup = 0.84), and the SUV (RSUV = 0.88), while that in the DTM-ELL is also strongly correlated with that in THUMS under impact by the family car (Rfamily-car = 0.76), the pickup (Rpickup = 0.79), and the SUV (RSUV = 0.73). Compared with the DTM-YLL, the correlation between the DTM-ELL and THUMS is lower, and the average peak stress in the femur of the DTM-ELL is only 62.64% of that in the DTM-YLL and 62.45% of that in THUMS. The cross-model comparison with THUMS demonstrates consistent biomechanical trends. It should be emphasized that THUMS is a computational benchmark rather than experimental ground truth, and because it represents a standard adult, lower agreement with the elderly model is expected. In addition, as shown in Figure 8, further simulations of the DTM-ELL were performed to estimate the impact forces and knee shear displacements under lateral impact by the three different car types at speeds of 30, 40, and 50 km/h, respectively. It can be observed that, for the same vehicle model in the CPC simulation, the higher the collision speed, the greater the impact force and knee joint shear displacement of the DTM-ELL, and the higher the corresponding risk of femur and knee joint injury. This result is consistent with accident data analyses, which show that injuries to the femur and knee joint are frequently observed when a pedestrian is struck by a passenger car.

### 3.3. Evaluation of the CPC Accident Reconstruction

**Figure 9:**
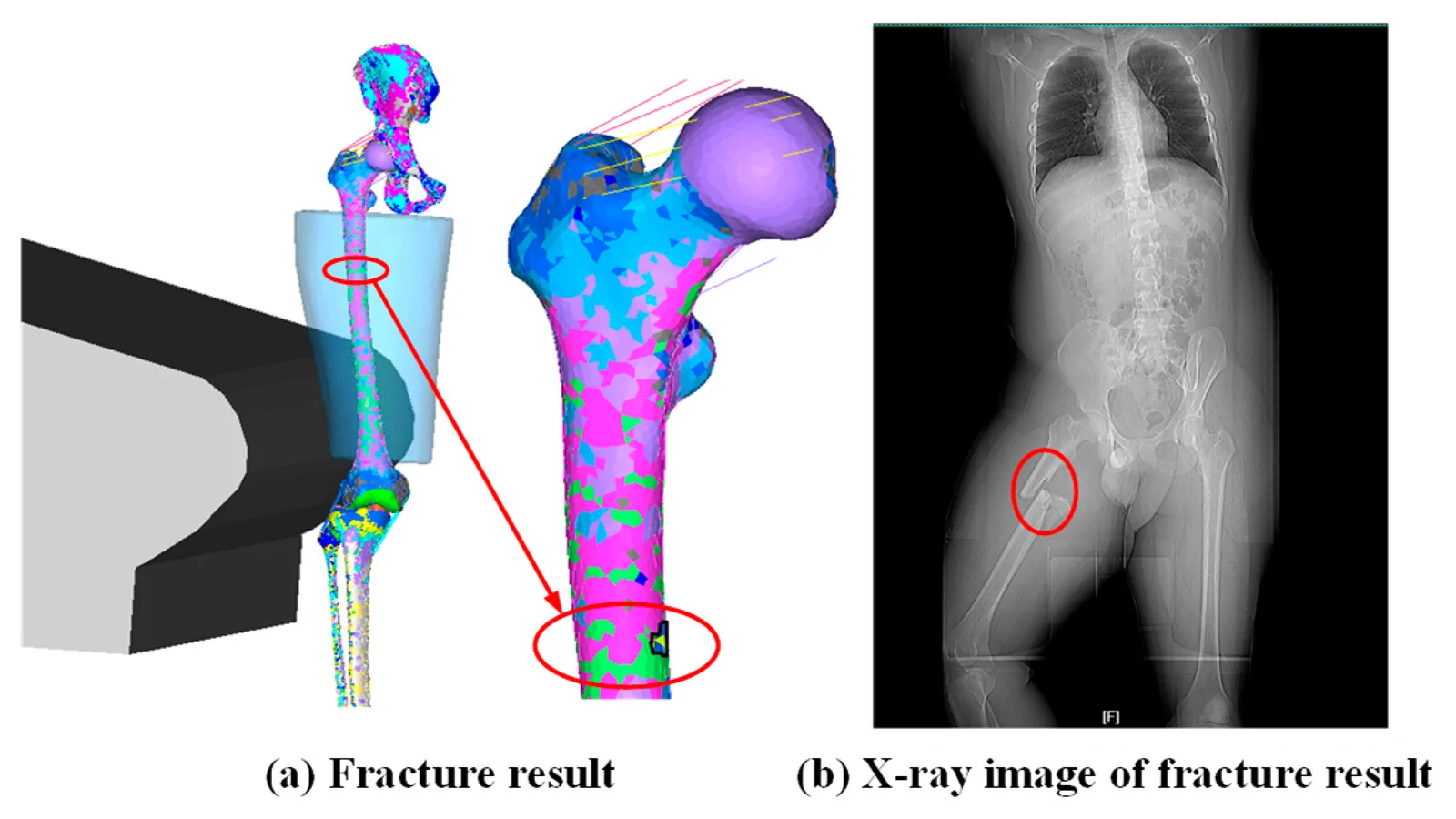
Comparisons of the bone fracture between FE simulation and X-ray image.

As shown in Figure 9a, the FE simulation based on the DTM-LL of the selected traffic accident patient shows that, after 3.1 ms of initial contact between the car and the human body, a fracture begins to appear in the proximal one-third of the femur, followed by expansion of the fracture crack, and ultimately the femur fractures completely, which is consistent with the femoral fracture obtained from the X-ray image of the patient, as shown in Figure 9b. Based on this result, a review of the CPC simulation process shows that when the front end of the car suddenly collides with the upper part of the knee joint, the hip joint remains stationary because of inertia, and the maximum bending stress on the femur increases over time. Finally, the outermost cortical bone reaches the effective stress threshold of 116 MPa at 3.1 ms, and the femur begins to show obvious cracks. The X-ray in Figure 9b shows that the fracture in the patient’s femur occurred in the proximal one-third of the right femur, and the fracture on the medial side is more severe than that on the lateral side of the femur. It is inferred that the femur began to fracture on the medial side, which is also consistent with the FE simulation results. Therefore, these results indicate that the model developed according to the modeling method described above can be used to study femur fractures in CPC.

## 4. Discussion

### 4.1. Comparison of Femoral Mechanical Properties Between DTM-ELL and DTM-YLL Due to Age

**Figure 10:**
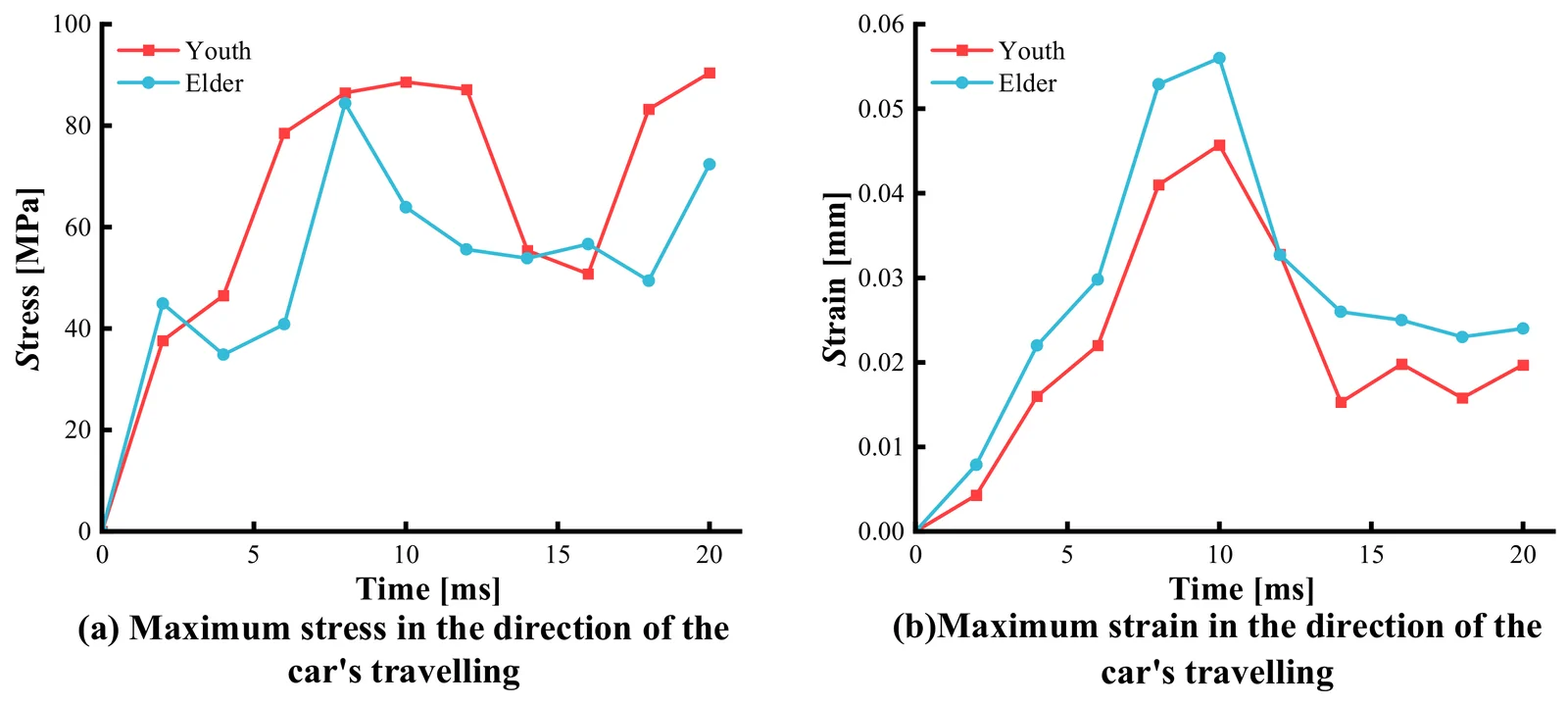
The real-time maximum stress and strain of the femurs in DTM-ELL and DTM-YLL impacted by the family-car at the speed of 40 km/h.

In the femoral three-point bending test and CPC simulation, we found that the bone stress of the DTM-ELL was lower than that of the DTM-YLL. With increasing age, Young’s modulus of human bones shows a decreasing trend, which has a marked effect on bone fracture and damage, but most researchers have not paid sufficient attention to this in CPC studies. Especially after the age of 70, the density and quality of human bone gradually decrease for a variety of reasons, and the bone microstructure deteriorates, leading to increased bone fragility and a reduced ability to withstand deformation. Figure 10 compares the maximum stress and strain of the femurs in the DTM-ELL and DTM-YLL under impact by the family-car model at a speed of 40 km/h, where the stress and strain of the femur were checked every 2 ms. It can be seen that the maximum stress of the DTM-ELL is generally lower than that of the DTM-YLL under the same impact load, whereas the maximum strain is greater, which is consistent with the conclusion reported by Zeng et al. [3]. Therefore, in the study of lower-limb bone fractures in the elderly, it is necessary to take into account the age-related degradation of bone material parameters such as Young’s modulus and bone mineral density. Especially in forensic identification, forensic experts should pay attention to the critical factor of age when dealing with lower-limb traffic accident injuries. These results suggest that age-related material property degradation and soft-tissue cushioning significantly modulate the femoral stress and strain distributions in the subjects studied, although other unquantified factors (such as BMI, osteoporosis severity, and impact posture) also play important roles. However, because only one subject per age group was evaluated (N = 1), these subject-specific findings cannot be directly generalized to population-level biomechanical patterns. Although the maximum average stress in the elderly femur was approximately 62.64% of that in the young model, lower nominal stress does not directly indicate a lower fracture risk. A structure with reduced stiffness exhibits lower stress under identical loads but undergoes greater local strain. Therefore, femoral injury vulnerability must be evaluated relative to tissue-specific failure thresholds rather than to stress magnitude alone.

### 4.2. Implications of Car Front Contour Design for the Femur Injury

In CPC, the masses of the family car, pickup, and SUV were 1.097 t, 2.711 t, and 1.51 t, respectively. A comparison between the family car and the SUV showed that the lighter family car caused more severe damage to the femur. To explore the reasons for this, we studied the global kinematic behavior of the lower limb impacted by the three cars, and found that as the impact progressed, the part of the femur above the impact point moved in the opposite direction of the vehicle’s movement relative to the front panel of the SUV because of inertia. However, the SUV model used in the FE simulation had a larger distance between the front bumper and the front panel than the family car, allowing more space for the femur to move during impact. Moreover, owing to the exterior design of the SUV hood, the part of the DTM-ELL above the hip joint first collided with the hood during impact, producing a pronounced anti-deformation effect on the femur and reducing the risk of femoral fracture.

**Figure 11:**
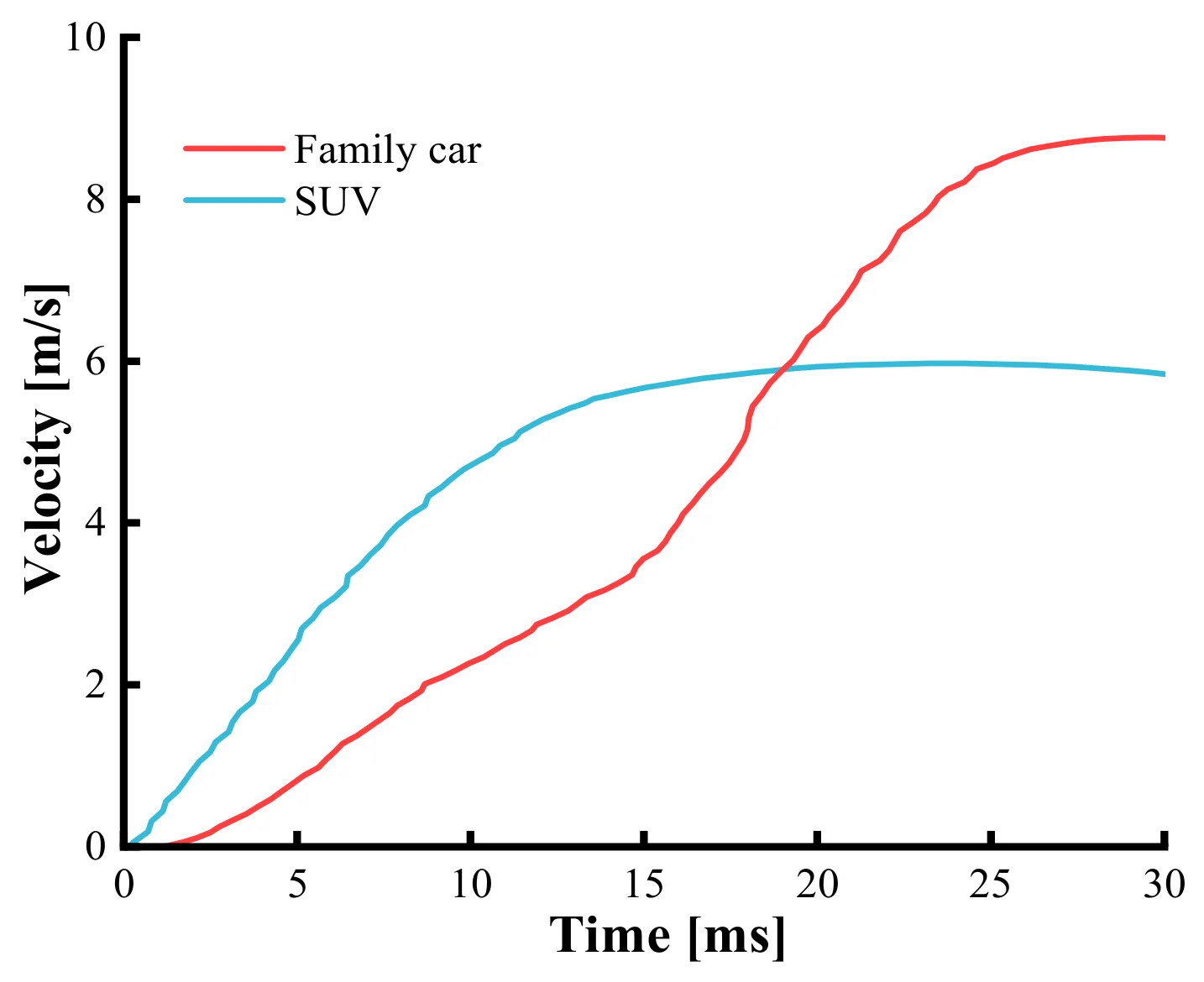
Femoral velocity along the driving direction under impacts of SUV and family-car at the speed of 40 km/h.

As shown in Figure 11, the femoral velocity, as the global kinematic behavior along the driving direction under impact by the SUV and family car at the same speed of 40 km/h, was compared. It can be seen that the femoral velocity increases faster during the first 15 ms of CPC because of the stronger impact momentum caused by the larger mass of the SUV. Subsequently, as shown in Figure 6, owing to the difference in front contour dimensions between the SUV and the family car, the leading edges of their hoods collide differently with the hip bone and proximal femur of the pedestrian. This difference causes the impact momentum of the SUV to be shared by the femur and hip bone, resulting in a pronounced decrease in the rate of change of the femoral velocity, whereas the impact momentum of the family car is still borne only by the femur, so that the rate of change of the femoral velocity does not decrease significantly but continues to increase. Ultimately, the continuous cumulative impact of the family car on the femur resulted in more severe femoral injury than that of the SUV. The present study focuses on femoral fractures of the elderly in CPC. Although the design of the front panel and bumper of the SUV used in this study can reduce femoral injury, this does not mean that this design reduces overall lower limb injury during the collision, because in the CPC process, in addition to femoral fracture, knee ligament damage and hip and tibiofibular injuries are also important lower-limb consequences, which will be studied in the future. It should be emphasized that the observed biomechanical variations and the reduction in femoral stress under SUV impact are specific to the three representative vehicle models evaluated in this study. These effects are influenced by a combination of vehicle mass, overall stiffness distribution, bumper height, and front-end geometry, rather than by the front-end contour alone. Broader conclusions regarding vehicle safety design would require evaluating a wider variety of vehicle models and structural configurations.

### 4.3. Contribution of Surrounding Soft Tissues to the Femoral Stress in CPC

**Figure 12:**
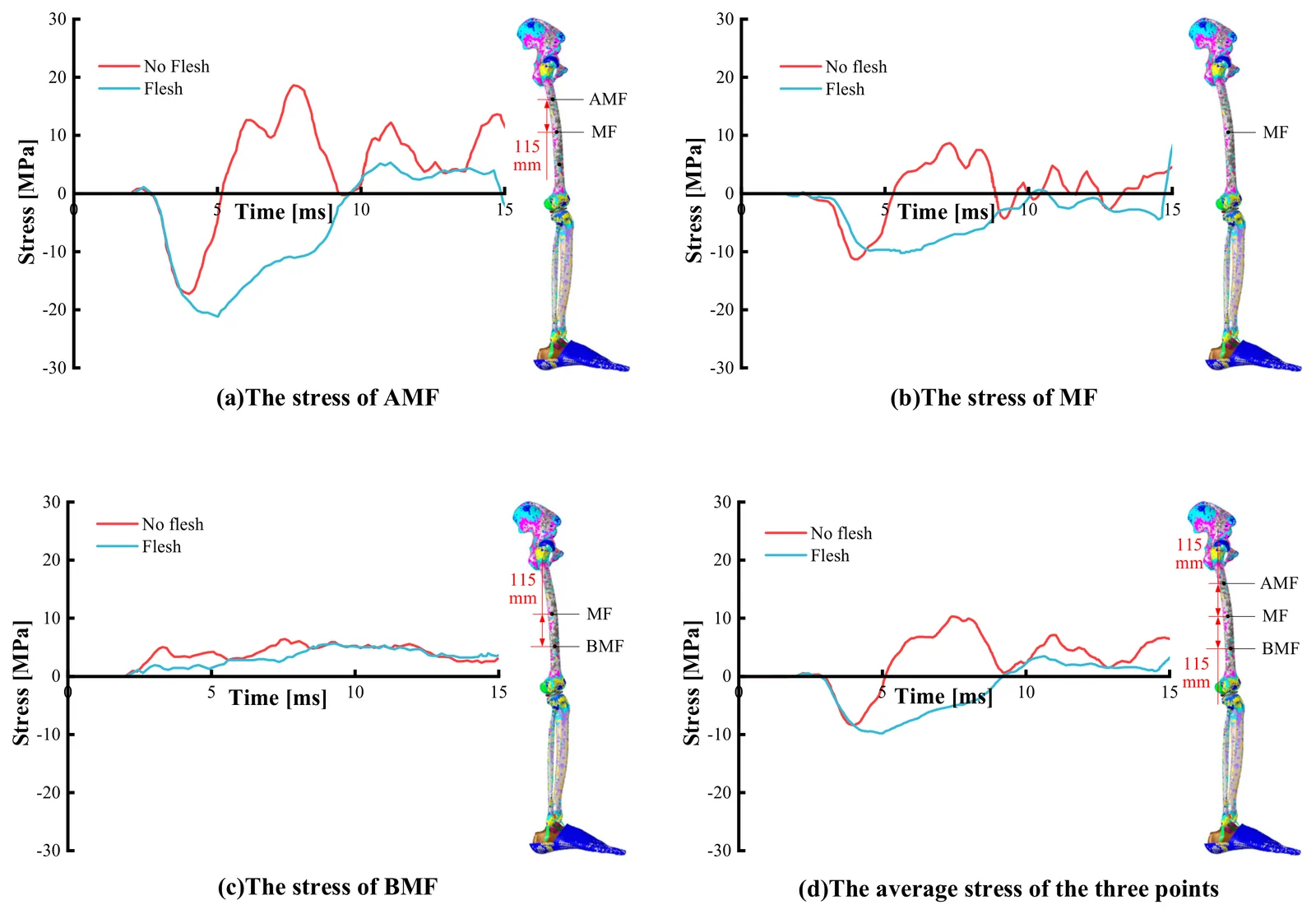
Real-time stress at three points on the femur in the DTM-ELL with and without surrounding soft tissues.

To investigate the role of the surrounding soft tissues during this process, a new DTM-ELL without surrounding soft tissues was developed to re-simulate CPC, and the results showed that the femoral fracture occurred at a distance of 1/3 from the distal end, and that a fracture appeared near the impact point 0.5 ms after contact between the car and the femur. Compared with the DTM-ELL with surrounding soft tissues, the DTM-ELL without surrounding soft tissues significantly shortened the time to crack initiation in the femur, and the fracture location also changed. When no muscle acts as a cushion, direct rigid contact between the car and the bone leads to an instantaneous increase in stress at the impact point, resulting in fracture. To analyze the degree of real-time cushioning provided by the surrounding soft tissues during CPC, we measured the stress at the midpoint of the femur (MF) and at two other points 115 mm above and below the MF (AMF and BMF) in the DTM-ELL with and without surrounding soft tissues, respectively. The curves of the real-time stress at the three points are shown in Figure 12. The comparison indicates that the surrounding soft tissues play a significant cushioning role, reducing the maximum femoral stress by 29.56% during the CPC process. The assumption of passive soft tissues during the immediate collision phase (0–20 ms) is justified because reflex muscle activation times in humans typically range between 100 and 200 ms, which far exceeds the primary impact duration. Furthermore, elderly pedestrians exhibit prolonged reaction times and delayed neuromuscular reflex responses, further supporting the validity of neglecting active muscle contraction during the initial 20 ms fracture window.

### 4.4. Limitations

In this paper, to study the mechanical response and injury of the femur, the DTM-LL was developed and validated using a variety of methods, yielding a finite element model of the lower limb that meets the requirements. However, the main work focused on fractures and injuries of the lower-limb femur in the elderly during the CPC process. Therefore, the DTM-LL was only modeled with a uniformly distributed force above the pelvis, simulating the loading effect of the upper body on the lower limb. Applying a simplified 400 N load above the pelvis neglects the rotational dynamics, inertia, and mass distribution of the complete torso during impact, which may influence hip translation, femoral bending moments, and knee shear responses. In the future, we will develop a full-body digital twin model of the Chinese 50th-percentile elderly for comparison with the DTM-ELL, and more biomimetic solid-element ligaments will be developed to study ligament damage during the CPC process.

In this study, the DTM-LL was developed and validated to evaluate the femoral mechanical response and impact injury under CPC conditions. However, the current model simplified the upper body contribution by applying a uniformly distributed 400 N load above the pelvis. This simplification neglects the rotational dynamics, inertia, and mass distribution of the complete torso during collision, which may influence hip translation, femoral bending moments, and knee shear responses. In future work, a full-body digital twin model of the Chinese 50th-percentile elderly will be developed, and more biomimetic solid-element ligaments will be incorporated to comprehensively evaluate whole-body kinematics and ligament damage mechanisms during CPC.

Furthermore, several other methodological limitations of this study should be explicitly noted. First, the models were developed from CT data of only one elderly and one young volunteer (N = 1 per group). Although termed a Digital Twin Model, it currently serves as an offline computational replica of the scanned volunteer without real-time sensor feedback. Due to substantial inter-individual anatomical variability, the findings reflect subject-specific biomechanical responses rather than population-level injury patterns. The elderly volunteer (age 65) and accident victim (age 62) represent the young-old demographic (61–70 years) of the elderly population. The 150-property empirical mapping based on HU values generates a continuous modulus spectrum (3439–15,224 MPa for elderly vs. 4028–17,815 MPa for youth). While slight numerical overlap exists at specific density boundaries, the elderly model exhibits an overall lower mean Young’s modulus and a thinner cortical shell. The conclusions should be interpreted as representative of the 61–70 age cohort rather than severe osteoporotic octogenarians and older. Second, the soft tissue was represented as a simplified, passive layer without incorporating active muscle behavior or detailed muscle contractions during collision. Third, bone material properties were assigned based on literature-derived relationships without patient-specific osteoporosis stratification or strain-rate-dependent bone formulations. Fourth, only lateral impacts under three fixed vehicle speeds were investigated. Variations in pedestrian posture, walking velocity, impact angle (frontal or oblique), and active braking posture were not systematically evaluated and will be addressed in future full-body modeling studies.

## 5. Conclusions

A DTM-ELL for the Chinese 50th-percentile elderly was developed based on CT images, in which 150 material properties were assigned to the bones according to Hounsfield units. The accuracy of the developed model was verified under various simulation conditions, and a CPC traffic accident was reconstructed with good simulation results. Therefore, the DTM-ELL developed in this study is applicable to the study of lower-limb femoral impact fracture injuries in the elderly. The FE simulation results show that age and surrounding soft tissues are two of the most important parameters affecting femoral impact fracture injuries of the elderly during the CPC process. With respect to age, the maximum average stress of the DTM-ELL femur is 62.64% of that of the DTM-YLL under the same CPC conditions. Surrounding soft tissues, acting as an effective cushion during the CPC process, reduce the maximum femoral stress by 29.56% compared with the model without surrounding soft tissues. In the future, we will continue to study CPC at a speed of 80 km/h to observe the biomechanical response of the lower-limb models under a low-probability high-speed impact.

## Data Availability

The original contributions presented in this study are included in the article.
